# Gluten-Free Diet Compliance in Children With Celiac Disease and Its Effect on Clinical Symptoms: A Retrospective Cohort Study

**DOI:** 10.7759/cureus.50217

**Published:** 2023-12-09

**Authors:** Assia Mouslih, Karima El Rhazi, Nassiba Bahra, Mounia Lakhdar Idrissi, Moustapha Hida

**Affiliations:** 1 Laboratory of Epidemiology, Clinical Research, and Community Health, Faculty of Medicine and Pharmacy, Sidi Mohamed Ben Abdellah University, Fez, MAR; 2 Department of Pediatric Diseases, Faculty of Medicine and Pharmacy, Hassan II Hospital, Fez, MAR; 3 Laboratory of Epidemiology and Health Science Research, Faculty of Medicine and Pharmacy, Sidi Mohammed Ben Abdellah University, Fez, MAR

**Keywords:** celiac diseases, celiac disease in children, children with celiac disease, children, children effect on clinical symptoms, compliance, celiac disease, gluten-free diet

## Abstract

A gluten-free diet (GFD) is the only scientifically proven treatment for celiac disease (CD). Strict adherence to this diet in children yields excellent results in terms of the clinical symptoms present at the time of diagnosis. Despite the constraints associated with following this diet, it remains the only hope for children with CD to have a better quality of life and life expectancy.

Methods: A retrospective descriptive cohort study was carried out on children diagnosed with CD in the pediatrics department of the Hassan II University Hospital in Fez, Morocco. The children were followed up for 18 months, during which time they were seen as outpatients at different frequencies depending on their clinical condition and degree of compliance with the diet.

Results: Only half of the diagnosed children continued to follow our structure. Compliance with the gluten-free diet varied from 58.7% (n = 84) of children who strictly followed the GFD to 3.5% (n = 5) of children who never followed the diet. Compliance was significantly correlated with the child's age, with adolescents being the least compliant (p = 0.03). Similarly, a correlation was observed between compliance with the diet and the disappearance of symptoms (p <0.01), the persistence of certain symptoms (p = 0.02), and the occurrence of complications (p = 0.01). The majority of children (87.3%) had their clinical symptoms resolved within a mean delay of 6.4±3.6 months, with a mode of three months. The speed of symptom resolution differed from one symptom to another but remained statistically correlated with the degree of GFD compliance (p = 0.03).

Conclusion: Despite the excellent results of a GFD on clinical symptoms in children, the discrepancies observed between compliance and non-compliance call for close follow-up of children with CD to avoid complications and repercussions on the vital prognosis in adulthood.

## Introduction

Celiac disease (CD) is a systemic immunologic disorder affecting multiple organ systems in genetically predisposed individuals, triggered by the ingestion of gluten-containing products [1, 2] and characterized by life-long gluten intolerance [3].

Around 2,000 years ago, the Greek physician Aretaeus provided the earliest clinical description of it [4]. In 1887, when Samuel Gee completed the discovery of the disease, he suggested that dietary treatment might be beneficial [5, 6]. However, it was only after World War II that Wim Dicke established the pathogenic link between gluten and the disease [5, 7]. Gluten is the main environmental factor responsible for the development of CD [8]. It is the antigenic protein mixture for CD [9], and the onset of clinical presentation occurs only when there is sufficient gluten in the diet [10].

The world of CD is rapidly changing [11]. The clinical manifestation has changed [12], and the expression has become highly eclectic [13] and broad [8], making the disease a clinical chameleon [14]. The diagnosis and presentation of CD have changed since its definition in the 1950s, but the gluten-free diet (GFD) remains the only scientifically proven effective treatment [9, 15].

The adoption of GFD should be initiated only after confirmed diagnosis [16, 17], as it is a lifelong commitment with challenges of compliance and barriers to access to gluten-free foods [18, 19], and most importantly, it is economically burdensome [19]. Children with CD should be monitored for symptom improvement, compliance, and quality of life [20]. This is more complex and restrictive than a normal diet [21].

After diagnosis, patients with CD should be referred to an expert dietitian. The GFD requires knowledge not only of hidden sources of gluten, as it is present in many sources in the diet [18, 22], but also of healthy gluten-free cereal alternatives [8]. This dietary treatment should be continued for life [7]. The first few weeks of GFD can already show clinical improvement [23]. Its effectiveness is most spectacular in symptomatic patients [15]. Besides the clinical improvement, this treatment helps to prevent bone and autoimmune complications [1, 24].

Dietary compliance is highly variable in patients with CD [25] and is influenced by dietary habits [26]. In our context, the implementation of the GFD is difficult because the Moroccan diet is based on a significant consumption of wheat and barley [4, 14, 27], and the GFD means a change in the eating habits of the Moroccan people [28]. However, if the patient shows no clinical response or partial response to gluten withdrawal, a careful evaluation is recommended to rule out accidental ingestion of gluten or poor compliance with the general diet [29]. The present study aimed to evaluate the impact of GFD compliance on the clinical symptoms and outcomes of children with CD.

## Materials and methods

Study design 

This is a retrospective cohort study conducted among 324 children diagnosed with CD and treated in the gastrointestinal unit of the Hassan II University Hospital in Fez, Morocco, between January 1, 2010, and December 30, 2019. The importance of this unit in the diagnosis and treatment of pediatric diseases justifies the choice of the research location. It also serves as a pediatric gastroenterology referral center, receiving patients from places outside the Fez-Meknes region.

Data collection

Data were gathered from the medical files of 324 children diagnosed with CD. The information gathered was as follows: (1) Socio-demographic data: gender, diagnosis age, place of residence (urban or rural), and the distance between the patients’ residence and the hospital calculated in kilometers; (2) Gluten effect before diagnosis: gluten introduction age and the gluten effect time, which is calculated in months and gives the time elapsed between the gluten introduction into the child's diet and the symptoms onset age for each child; (3) Clinical symptoms at the time of diagnosis: these are the symptoms recorded before the start of GFD treatment.

Celiac disease has a very wide spectrum of clinical manifestations, ranging from silent, atypical, classical, to severe forms (Table 1).

**Table 1:** The large spectrum of clinical manifestations of celiac disease (CD)

Clinical forms	Description
Silent CD	Characterized by a positive serologic test and villous atrophy, which confirms the disease. Generally, the silent form has been observed in high-risk populations (such as insulin-dependent diabetics and first-degree relatives).
Classic symptomatic	Includes diarrhea, vomiting, abdominal pain, bloating, anemia with malabsorption syndrome, and growth delay. Growth delay includes weight and height delay, weight loss, and weight stagnation.
Atypical/extra digestive	Manifests in numerous forms, ranging from an isolated growth delay to manifestations that may affect several organ systems and are sometimes related to an associated pathology.
Severe form	Celiac crisis is a constellation of severe diarrhea, weight loss, hypocalcemia, and hypoproteinemia. This fatal condition requires intensive care.

The diagnosis is based on clinical symptoms, positive serology, and an anatomopathologic study confirming villous atrophy. Once the diagnosis is made, the only current treatment is a GFD, but this is often difficult to follow and presents many difficulties. Dietary support is necessary to provide clear, detailed explanations and to ensure follow-up that focuses on symptom improvement, dietary compliance, and life quality. Dietary counseling helps to emphasize the importance of the complete elimination of gluten from the diet for life.

Once the diagnosis of CD is confirmed, nutritional deficiencies are assessed (the most common deficiencies are iron, vitamin D, potassium, sodium, and calcium), and bone age is determined by X-rays of the hand and wrist using the Greulich and Pyle method (one of the most appropriate and rapid methods for determining bone age) [30], and monitoring is done for the onset of associated diseases in diagnosed children.

Compliance analysis of GFD

The aim of the follow-up was to assess each child's GFD compliance after a CD diagnosis. For 18 months, we retrospectively followed our population to assess their GFD compliance. Follow-up was carried out routinely at six, 12, and 18 months, unless unforeseen.

Therapeutic compliance is defined as the degree of adequacy between the patient's behavior and therapeutic recommendations. Responses were classified according to the frequency of dietary deviations (voluntary or accidental consumption of gluten). This frequency is classified and interpreted according to a "gluten consumption frequency scale" designed to minimize the subjectivity of responses since compliance was assessed according to self-reporting by children with CD and their parents (Table 2).

**Table 2:** The frequency of compliance with a gluten-free diet (GFD)

Frequency	Compliance type
Always (strict compliance to the diet)	Good GFD compliance
Sometimes (consumes gluten once a month)	
Often (consumes gluten at least once a week)	Poor GFD compliance
Rarely (adheres to the diet occasionally)	

Effect on clinical symptoms of the GFD

The GFD effect is defined as clinical improvement if the clinical symptoms present at diagnosis disappear after the initiation of GFD. Non-response is defined as refractory CD when symptoms persist despite good GFD compliance for at least 12 months. In addition, the time to clinical improvement is calculated as the number of months from the onset of GFD to the resolution of all clinical symptoms and the catch-up of growth retardation.

Statistical analysis

The collected data were analyzed using IBM SPSS software version 26 (IBM Corp., Armonk, NY). Descriptive statistics were expressed as mean±standard deviation, median, and percentage. In our study population, we looked for a statistical relationship between the explanatory variables and the resolution of clinical symptoms and adherence to the GFD. The test of independence between the variables of interest and the explanatory variables was performed using chi-square (Chi2). The association was considered statistically significant if the p-value was <0.05. A Kaplan-Meier survival analysis was performed in our sample to determine the time to resolution of clinical symptoms as a function of GFD compliance.

Ethics

In keeping with the ethical principles of the Helsinki Declaration, the protocol of our study was submitted to the ethics committee of the Faculty of Medicine and Pharmacy of Hassan II Hospital, Fez, Morrocco. Approval was granted to proceed with the study (approval number: 12/22).

## Results

Sociodemographic characteristics

In total, 324 children were diagnosed with CD based on their clinical profile, and serologic, and histologic tests. Of both sexes, 60.8% were female (n = 197), and the remaining 39.2% were male (n = 127). The mean diagnosis age was 73.79±46.86 months, ranging from six to 204 months, of which 62% (n = 201) were diagnosed in childhood (Table 3)

**Table 3:** Distribution of children with celiac disease by age group and gender p-values <0.05 are considered significant

Age range	Female N(%)	Male N(%)	Total N(%)	P-value
Early childhood (6 months to 24 months)	27 (8.3)	20 (6.2)	47 (14.5)	0.03
Childhood (25 months to 120 months)	114(35.2)	87 (26.8)	201 (62)	
Adolescence (121 months to 204 months)	56(17.3)	20 (6.2)	76 (23.5)	
Total	197 (60.8)	127 (39.2)	324	

Of the study participants, 86.4% (n = 280) were urban residents, and the mean distance between their residence and the hospital center was 75.7±108.1 km (Table 3).

In our study, only 59.6% (n = 193) of diagnosed children were followed up during the study period. Children were lost to sight either immediately after diagnosis or later during the follow-up period.

Gluten effect before diagnosis

The mean age of gluten introduction was 6.4±2.1 months, with a range from three to 18 months. The mean gluten effect time was 35.4±34.4 months, ranging from 0 months to 13.5 years. In 8% (n = 13) of children, the gluten introduction triggered immediate symptoms. However, no significant association was found between the age of gluten introduction and the symptom onset age (p = 0.1). The mean gluten effect time was longer in females (39.0±39.5 months) than in males (30.8±26.0 months).

Clinical symptoms at diagnosis

In our study, the clinical symptoms were divided into classic (70.1%; n = 227), atypical (19.8%; n = 64), severe (7.1%; n = 23), and asymptomatic (3.1%; n = 10). Growth delay (58.6%; n = 190) and diarrhea (57.1%; n = 185) were the most frequent symptoms. At the time of diagnosis, 15.7% (n = 47) had a single symptom, while 75.4% (n = 226) had two to four digestive symptoms at the same time, and 1.7% (n = 5) had six symptoms. All children diagnosed with CD systematically benefited from dietary counseling. Nevertheless, only 59.6% (n = 193) of the children diagnosed in the pediatric department continued their follow-up in our structure.

Clinical evaluation at diagnosis

In addition, type 1 diabetes (T1D) was the most common associated disease (72.2%; n = 26), followed by hypothyroidism (13.9%; n = 5). Among the children in whom CD was associated with diabetes, only 27% (n = 7) of children developed T1D after initiation of GFD.

Nutritional status was assessed in 57.4% (n = 186) of diagnosed children, and micronutrient deficiencies were found in 86.6% (n = 161) of them. The most common deficiency was vitamin D deficiency (88.8%; n = 95), followed by iron deficiency (73.5%; n = 75) and hypocalcemia (53.5%; n = 54). Although CD doesn’t require medical treatment, 84.4% (n = 92) of children with deficiencies were taking supplements to correct them. As part of the assessment of the impact of celiac disease, an X-ray was performed for 48 children; 68.7% (n = 33) showed bone delay, the mode of delay of which was -2 years (30.3%; n = 10), with a maximum delay of -6 years (6%; n = 2).

Gluten-free diet compliance

**Figure 1:**
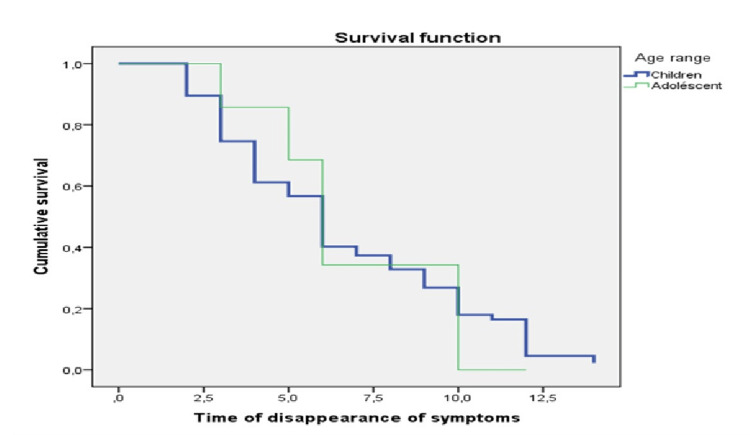
Analysis of the time of disappearance of symptoms as a function of age range The age range and the duration until the symptoms go away are expressed in months.

In our cohort, we had 84.6% (n = 121) with good GFD compliance, while 3.5% (n = 5) did not follow the diet despite CD. In this study, no association was observed between gender, place of residence, and diet compliance. However, compliance was significantly associated with children's age (p = 0.03), with more children than adolescents following the diet (Figure 1), the presence of an autoimmune disease at the time of diagnosis (p = 0.05), and the type of clinical symptoms present (p = 0.006).

Thus, the symptom-by-symptom correlation revealed that only children with delayed weight growth (p <0.01) and dehydration (p <0.01) were more likely to adhere to the diet.

Effect on clinical symptoms of GFD compliance

In the follow-up of our cohort, clinical improvement was obtained over a mean of 6.4±3.6 months, with a mode of three and a median of six months. Even though the mean time from gluten introduction to symptoms onset was long in comparison to the time from gluten exclusion to symptoms disappearance, no association was observed between the gluten effect time and the effect of GFD on clinical symptoms.

The time of clinical improvement varied from one symptom to another, ranging from adhering for 2.2 months (delayed walking) to a mean duration of 12.7 months (pallor) (Table 4).

**Table 4:** The mean duration of the clinical improvement after adhering to a gluten-free diet SD: standard deviation

Symptoms	Mean duration ±SD (months)
Delay in walking	2.2±1.7
Flu syndrome	2.5±1.9
Weight stagnation	3.5±1.7
Anorexia	5.8±3.8
Abdominal pain	6.1±3.5
Growth delay	6.2±3.7
Diarrhea	6.2±3.4
Lack of appetite	6.2±3.4
Constipation	6.3±3.7
Delayed puberty	6.4±3.6
Transit disorder	6.4±3.8
Bloating	6.4±3.5
Skin mycosis	6.4±3.6
Weight loss	6.6±3.8
Alteration of the general state	7.1±4.2
Anemia	7.2±4.2
Vomiting	7.3±3.5
Fever	7.3±3.0
Malnutrition	7.5±6.3
Urination burns	7.5±6.3
Asthenia	7.6±3.5
Oedemas	7.7±4.0
Muscular hypotonia	8.1±8.4
Epigastralgia	10.2±4.5
Pallor	12.7±8.3

During follow-up, refractory CD was detected in eight (5.5%) children enrolled in the GFD.

**Figure 2:**
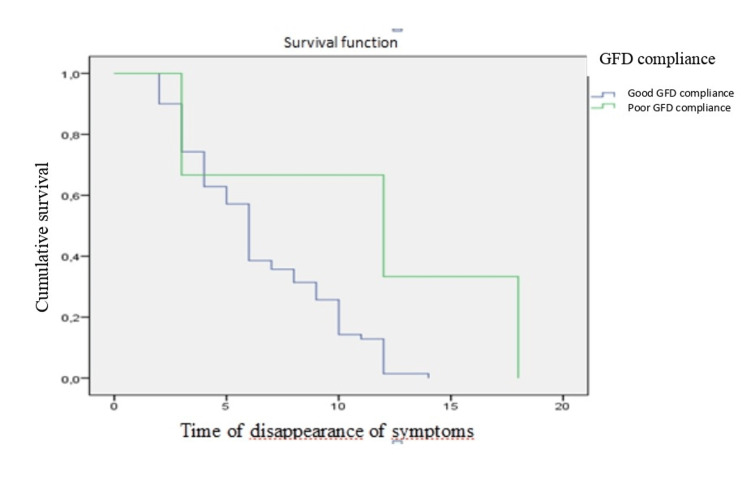
Analysis of the time the symptoms disappeared as a function of the gluten-free diet (GFD) compliance The time of disappearance of symptoms is expressed in months.

The GFD compliance was statistically significantly correlated with clinical symptom resolution (p = 0.01), refractory CD (p = 0.02), and even complication occurrence (p = 0.01), and the GFD effect was examined using McNeimar's survival curve. This curve compares dietary compliance with clinical improvement. The speed at which clinical symptoms disappeared increased significantly with GFD compliance (p = 0.03) (Figure 2).

## Discussion

A GFD continues to be the cornerstone of CD treatment to this day [31] and helps to improve clinical symptoms [32]. Prescribing the diet is straightforward, but its adoption and maintenance pose real challenges, particularly in developing countries [33, 34]. In our study, we selected a cohort of 324 patients diagnosed in the pediatric ward to assess adherence to the diet and its impact on clinical improvement.

Our study showed a low follow-up rate, similar to a study conducted in a tertiary pediatric referral center [35], where only 42.7% continued their follow-up. Similarly, a recent study in Minnesota estimated that less than half of patients with CD were followed up one year after diagnosis, and only one-third had their compliance assessed [36]. Even in adults, a study by Richter et al. [37] found that 25% were lost to follow-up after diagnosis. This confirms the difficulty of follow-up in CD [37] and forces more attention to be paid to the follow-up of children on GFD [38] since follow-up improves GFD compliance [39].

Even though gluten is an essential environmental factor in the onset of CD [40], the influence of gluten introduction on the onset of CD has been the subject of much debate [41]. Our study showed no association between the time of gluten introduction and the onset of symptoms. In The Environmental Determinants of Diabetes in the Young (TEDDY) study, the time of first introduction of gluten was not a risk factor [42]. Consequently, the timing of gluten introduction had no protective effect [43], and even the late introduction of gluten does not alter the risk of celiac disease in at-risk infants [44]. Similarly, in the European PreventCD study, mean daily consumption was not associated with an increased risk of CD [45]. This finding was corroborated by a prospective multicenter trial, which found no association between newborn exposure to gluten and the risk of developing CD [44]. This confirms that primary prevention is not possible for CD [46].

Clinical symptoms at diagnosis

In our study, the most diagnosed form is the classic form. The classic presentation is no longer the most common [47], but the best known [48]. The Marrakech series showed that the diagnosis is limited to the classic form [49], while another study conducted in southwest Iran showed that the classic form is the most dominant [50]. Other studies have increased the predominance of the classical form [51, 52]. In general, diagnosis in developing countries is based on symptomatic and classic forms [53]. Despite great variability from one country to another, the classic form of CD remains a common presentation in the pediatric age group [54].

The coexistence of CD with other autoimmune diseases is also classical [55]. The reason for this association has never been fully elucidated [56]. The most common associated disease is T1D [57, 58], with both diseases sharing common genetic material [59]. This predominance of T1D was confirmed in our study. Type 1 diabetes may be a precipitating factor in CD [59]. However, the influence of CD on the development of T1D remains controversial [60].

The effect of GFD on the development of autoimmune diseases has not been demonstrated [32, 61], but a study by Cosnes et al. [61] showed a protective effect of diet on the development of autoimmune diseases. Another multicenter study conducted in Italy demonstrated an association between GFD and a reduced risk of autoimmune disease [15] and helped prevent autoimmune complications [24].

However, the influence on the long-term development of diabetic complications is still unknown [33, 62]. Even in our study, there was no association between compliance with GFD and the development of T1D. However, GFD brings clinical improvements to diabetic children with CD [63, 64] and can influence insulin sensitivity [63].

Patients with CD often suffer from nutritional deficiencies [1]. The most common deficiencies are iron, vitamin D, and calcium [1,65]. The frequency of these deficiencies is due to intestinal malabsorption due to the involvement of the proximal small intestine [65-67]. This confirms our findings that our population is deficient in vitamin D, iron, and calcium.

This malabsorption of calcium and vitamin D has important pathophysiological roles in the development of osteoporosis [9, 68-70], but there is no conclusive data on the pathogenesis of bone involvement in CD [54]. In our study, osteoporosis was detected in 10.8% of cases, which is low compared to the prevalence of osteoporosis in children with CD, which varies from 15% to 25% in children with CD [71]. This low prevalence of osteoporosis in children with CD may be explained by the fact that the search for osteoporosis after diagnosis is not systematic in our context.

Gluten-free diet compliance

A strict diet excluding all gluten-containing foods [24, 52] is the only treatment for CD [51, 57]. In addition to its therapeutic role, it helps confirm the diagnosis [17, 72, 73] and completely eliminate the symptoms of the disease [74]. Despite the spectacular results of the GFD [15, 75], particularly in children [15], and its relative simplicity [15], in practice, it is restrictive, difficult to follow [15, 16], and presents real challenges [74].

Compliance with the diet varies considerably among people with CD [25]. Around half of patients with CD report adherence to the diet [75]. In general, compliance is estimated at between 45% and 80% [76]. In our population, compliance ranged from 58.7% to 84.6%. Similarly, the Marrakech study estimates that half of the patients were good observers [28] and the Constantine study shows that 78% of Algerians had good compliance [77].

Our result is high compared with a French study in which only 50% of patients with the disease adhered to the diet [78]. In Europe, compliance rates vary from 44% to 97% [75]. Butterworth et al. found good adherence to the GFD, with a compliance rate of 74% in Caucasians and 66.6% in South Asians [78]. Compliance is also associated with age in our study, which is similar to that reported by Leffler [76], where adolescents have compliance difficulties [32, 39, 57, 75]. In contrast, a study of adolescents in Italy showed a strict compliance rate of 72% [9].

Good GFD compliance leads to rapid clinical improvement [22, 24, 79], enhances quality of life [80, 81], reduces the occurrence of complications [14, 75], and guarantees a normal life expectancy [82]. This clinical improvement after GFD is used to assess the effectiveness of the treatment [17, 22, 24]. Clinical improvement is observed in 70%-95% of patients with CD [65]. This significant improvement can be observed within 48 hours, although it can take weeks or months to achieve clinical remission [1]. The results of our study are consistent with the literature, where 85.7% of patients showed clinical improvement with the disappearance of symptoms after following the diet. Clinical improvement was rapid, ranging from five days to five weeks [22, 24, 83].

Although the time to clinical resolution in our population seems longer than that reported in several studies [8, 9, 22, 24], the MRI study [15] showed that a few days of GFD corrected behavioral problems, loss of appetite, and weight gain within the first few weeks [15]. Symptoms did not have the same tendency to disappear after GFD [84]. However, improvements in nutritional status and body composition were achieved within the first year of strict adherence to the diet [19].

According to the World Health Organization, non-compliance is a major clinical problem in the management of patients with chronic diseases. Rates of non-compliance can vary from 15% to 93%, with a mean rate estimated at 50% [85]. Dietary non-compliance is a concern for caregivers [86], with total non-compliance reported by 6% to 37% of caregivers [17]. In our population, total non-compliance was observed in only 3.5% of cases. However, a high incidence of 50% has been reported for gluten consumption during GFD [6, 50, 80, 87].

Clinical sensitivity varies considerably from patient to patient. Some cannot tolerate traces of gluten, while others seem to tolerate large amounts [87]. The amount of gluten that can be tolerated without triggering an immune reaction has not been determined and varies from patient to patient [88]. This lack of dietary compliance is mainly due to the frequent presence of gluten in the food industry [15], the prominence of wheat in the diet [15], the high cost of gluten-free substitutes [8], and the ambiguity of labeling [89]. Compliance is also influenced by dietary habits [26] and the social context in developing countries [54, 55].

Limitation

Our study supports GFD compliance as an effective treatment, although it has limitations due to its retrospective nature and the survey instrument, which limits the collection of certain data on children's perceptions of the GFD and the psychometric view of clinical improvement. In addition, a significant number of children diagnosed in the gastroenterology unit were not followed up in our institution.

## Conclusions

Our study confirms that good compliance with GFD leads to rapid clinical improvement and the resolution of symptoms. This improvement allows both confirmation of the diagnosis and tertiary prevention by limiting the onset of complications and autoimmune diseases.

However, in our context, the follow-up of diagnosed children remains inadequate, and long-term compliance is a challenge for patients and healthcare professionals alike. Given the difficulty of changing dietary habits in a country where the diet is based on wheat and there is a scarcity and high cost of gluten-free products, a personalized follow-up and care plan for each patient is essential, which could be facilitated by new technologies.
